# Characterization of the rhesus macaque (*Macaca mulatta*) scrub typhus model: Susceptibility to intradermal challenge with the human pathogen *Orientia tsutsugamushi* Karp

**DOI:** 10.1371/journal.pntd.0006305

**Published:** 2018-03-09

**Authors:** Piyanate Sunyakumthorn, Suwit J. Somponpun, Rawiwan Im-erbsin, Tippawan Anantatat, Kemajittra Jenjaroen, Susanna J. Dunachie, Eric D. Lombardini, Robin L. Burke, Stuart D. Blacksell, James W. Jones, Carl J. Mason, Allen L. Richards, Nicholas P. J. Day, Daniel H. Paris

**Affiliations:** 1 Department of Veterinary Medicine, United States Army Medical Directorate, Armed Forces Research Institute of Medical Sciences (USAMD-AFRIMS), Bangkok, Thailand; 2 Mahidol Oxford Tropical Medicine Research Unit, Mahidol University, Bangkok, Thailand; 3 Nuffield Department of Medicine, Oxford University, Oxford, United Kingdom; 4 Viral & Rickettsial Diseases Department, Naval Medical Research Center, Silver Spring, MD, United States of America; 5 Preventive Medicine and Biostatistics Department, Uniformed Services University of the Health Sciences, Bethesda, MD, United States of America; 6 Department of Medicine, Swiss Tropical and Public Health Institute, Basel, Switzerland; 7 Faculty of Medicine, University of Basel, Basel, Switzerland; University of Tennessee, UNITED STATES

## Abstract

**Background:**

Scrub typhus is an important endemic disease in tropical Asia caused by *Orientia tsutsugamushi* for which no effective broadly protective vaccine is available. The successful evaluation of vaccine candidates requires well-characterized animal models and a better understanding of the immune response against *O*. *tsutsugamushi*. While many animal species have been used to study host immunity and vaccine responses in scrub typhus, only limited data exists in non-human primate (NHP) models.

**Methodology/Principle findings:**

In this study we evaluated a NHP scrub typhus disease model based on intradermal inoculation of *O*. *tsutsugamushi* Karp strain in rhesus macaques (n = 7). After an intradermal inoculation with 10^6^ murine LD_50_ of *O*. *tsutsugamushi* at the anterior thigh (n = 4) or mock inoculum (n = 3), a series of time course investigations involving hematological, biochemical, molecular and immunological assays were performed, until day 28, when tissues were collected for pathology and immunohistochemistry. In all NHPs with *O*. *tsutsugamushi* inoculation, but not with mock inoculation, the development of a classic eschar with central necrosis, regional lymphadenopathy, and elevation of body temperature was observed on days 7–21 post inoculation (pi); bacteremia was detected by qPCR on days 6–18 pi; and alteration of liver enzyme function and increase of white blood cells on day 14 pi. Immune assays demonstrated raised serum levels of soluble cell adhesion molecules, anti-*O*. *tsutsugamushi*-specific antibody responses (IgM and IgG) and pathogen-specific cell-mediated immune responses in inoculated macaques. The qPCR assays detected *O*. *tsutsugamushi* in eschar, spleen, draining and non-draining lymph nodes, and immuno-double staining demonstrated intracellular *O*. *tsutsugamushi* in antigen presenting cells of eschars and lymph nodes.

**Conclusions/Significance:**

These data show the potential of using rhesus macaques as a scrub typhus model, for evaluation of correlates of protection in both natural and vaccine induced immunity, and support the evaluation of future vaccine candidates against scrub typhus.

## Author summary

Scrub typhus is a febrile illness caused by bacteria that invade and live within cells of the immune and blood vessel systems. Small earth-bound mites can bite humans and transmit these bacteria into the skin. Scrub typhus is treatable with antibiotics, but currently there is no scrub typhus vaccine available. Unfortunately if humans get scrub typhus, the immune response is usually weak and short-lived, especially against different strains, and affected individuals can get ill again within a year. This is a problem in areas where the infection is very common and a vaccine could be an effective approach to protect susceptible humans against scrub typhus.

In this study, we characterized the immune response and disease features of scrub typhus in rhesus macaques by inoculating the bacteria directly into the skin–similar to the mite bite in nature–previously this had only been done in cynomolgus macaques (*Macaca fascicularis*). We found that the scrub typhus symptoms and immune responses of rhesus macaques resemble more closely the human responses than those of cynomolgus macaques. Studying the immune response in rhesus macaques will help us to understand how humans react against different bacterial proteins, to identify new markers of protection and to find the strongest vaccine candidates. This will then help us develop new and better vaccines (and also diagnostics) against scrub typhus in the future.

## Introduction

Scrub typhus is a common but under recognized acute febrile illness caused by *Orientia tsutsugamushi*, a Gram-negative obligate intracellular bacterium. Mites (Acari: Trombiculidae), serve as both the vector transmitting the bacteria during their larval stage (chigger) to vertebrate hosts and the reservoir maintaining the bacteria during their life cycle [1]. The clinical signs and symptoms of scrub typhus are similar to other infectious diseases, which complicates clinical diagnosis. Despite available drugs (tetracyclines, chloramphenicol, azithromycin, rifampicin), scrub typhus remains an underappreciated health care problem due to difficulties in clinical and laboratory diagnosis, delayed treatment responses in northern Thailand and southern India, and the lack of an effective vaccine [2].

Studies of scrub typhus pathogenesis and immunity, critical to vaccine development, require suitable animal models to understand the interaction and temporal dynamics between *O*. *tsutsugamushi* and host responses. Various animal models have been developed for scrub typhus, including mice, guinea pigs, and non-human primates (NHP) [3–7]. In mouse models, both inbred and outbred mice developed clinical manifestations in various degrees; however, the mouse models did not develop reproducible pathology resembling human scrub [8]. In immunological studies, a CD8 T-cell mediated cytotoxic immune response was shown necessary for clearance of *O*. *tsutsugamushi* and protection from lethal infection [9, 10]. Although cell-mediated immunity (CMI) plays a central role in heterologous protection amongst the different strains of *O*. *tsutsugamushi* in inbred mice it was short-lived and waned after few months [11, 12]. Recently, apoptosis was proposed as a potential contributor to this phenomenon in humans, but this alone does not explain all findings associated with the transience of broad immune protection [11, 12]. A NHP model mimicking human disease will enable investigations into these mechanisms [4, 13].

Previous studies in NHPs; Chattopadhyay *et al*. evaluated a truncated recombinant 56-kDa outer membrane protein of the Karp strain (Kp r56) as a vaccine candidate in cynomolgus monkeys *(Macaca fascicularis)*, which induced both cellular and humoral immune responses that decreased local inflammation at the inoculation site, but did not confer protection to re-infection [3]. Then, Walsh *et al*. characterized the eschar and regional lymph node using histological and immunohistochemistry staining and demonstrated the localization of *O*. *tsutsugamushi* within the eschar and a regional lymph node. However, only two out of six monkeys developed necrotic eschars with unequivocal black crusted ulcers at the injection site [14]. A recent vaccine study using 47-kDa *htrA* candidate vaccines in cynomolgus monkeys demonstrated induction of sterile immunity against high-dose homologous challenge of *O*. *tsutsugamushi*. In addition, Paris *et al*. provided the first phenotypic correlates of immune protection in scrub typhus and presented the time course dynamics of bacteremia and host immune responses [4, 5, 13, 14]. Clearly further detailed studies of natural and vaccine-induced immune mechanisms in response to *O*. *tsutsugamushi* are required, with evaluation of other vaccine targets. Colony-reared cynomolgus monkeys are scanty compared to rhesus macaques (*Macaca mulatta*) which are more widely used as infectious disease models, and are likely to have more immunological data available. Even though cynomolgus macaques are considered suitable NHP models for scrub typhus, the disease manifestations are mild and correlates of immune protection appear delayed compared to humans. The aim of this study was to evaluate the role of rhesus macaques for a standardized NHP disease model for scrub typhus, in view of dissecting the dynamics of disease dissemination and early host-pathogen interactions in both natural infection and the vaccination/challenge setting with further characterization of early innate and adaptive immune responses. We anticipated that this study would provide improved data on the factors/parameters associated with immune protection and provide an animal model with sufficient parallels to humans for evaluating the safety, immunogenicity and efficacy for future scrub typhus vaccine candidates.

## Material and methods

### Ethics statement

All animal research was performed strictly under approved Institutional Animal Care and Use Committee (IACUC) protocol by the IACUC and Biosafety Review Committee at the Armed Forces Research Institute of Medical Sciences (AFRIMS) Bangkok, Thailand, an AAALAC International-accredited facility. The IACUC protocol number was PN12-01 (approved 31st Jan 2012). The animal research was conducted in compliance with Thai laws, the Animal Welfare Act, and all applicable U.S. Department of Agriculture, Office of Laboratory Animal Welfare and U.S. Department of Defense guidelines. All animal research adhered to the Guide for the Care and Use of Laboratory Animals, NRC Publication (8^th^ Edition) [15].

Animals were housed individually in standard squeeze-type stainless steel cages with a minimum floor space of 4.4 square feet equipped with standard enrichments and exposed to ambient environmental conditions inside an Animal Biosafety Level 3 (ABSL-3) containment laboratory. All NHPs were fed daily with commercially prepared old-world primate extruded feed and supplemented with fresh fruit or vegetable four times per week. Fresh chlorinated water (5–10 ppm) was provided *ad libitum* via automatic water valves. Cages were cleaned daily and sanitized biweekly. Animals were trained for 2–3 weeks for pole-collar-chair restraint prior to the commencement of the study in which no anesthesia was required. All other procedures were performed under anesthesia using ketamine hydrochloride, and all efforts were made to minimize stress, improve housing conditions, and to provide enrichment opportunities. Animals were euthanized by ketamine hydrochloride injection (5–20 mg/kg intramuscularly) followed by barbiturate (86.7 mg sodium pentobarbital/kg) in accordance with the Guidelines for the Euthanasia of Animals (2013 Edition of the American Veterinary Medical Association).

### Non-human primates

Laboratory-reared Indian-origin rhesus macaques (*Macaca mulatta*) from AFRIMS colony were used in this study. The AFRIMS rhesus macaque colony was established in 1981. The Indian-origin rhesus macaques were originally imported from primate centers in the USA and have been reared at the AAALAC International-accredited AFRIMS facilities since 1999. All monkeys utilized in this study were born and reared at AFRIMS. Six of 7 macaques had participated in a prior malaria study (*Plasmodium cynomolgi*) and one was naïve. The seven macaques (3 males and 4 females) were three years of age and weighed between 4.7–5.4 kg at the start of the study. The animals had no prior exposure to *O*. *tsutsugamushi* with negative antibody titers to *O*. *tsutsugamushi* and negative serology for Simian Immunodeficiency Virus (SIV), Simian Retrovirus (SRV), Simian T-lymphotropic Virus (STLV-1) and *Macacine Herpesvirus* 1 (B virus). The macaques were randomly allocated to either a ‘control or mock infection’ (n = 3) or ‘*O*. *tsutsugamushi* infection’ (n = 4) group. One week prior to Inoculation Day 0, macaques underwent a complete physical examination that included a blood draw (total of 5.0 ml of whole blood) for complete blood count, blood chemistry analysis, *O*. *tsutsugamushi*-specific quantitative real-time polymerase chain reaction (qPCR) assay, and immunological and serological tests to establish baseline values. All animals had good general health as determined by the attending veterinary staff through complete physical examination and laboratory examinations (completed blood count (CBC) and blood chemistry).

### *Orientia tsutsugamushi* Karp inoculum

The human isolated strain (Karp, Papua New Guinea, 1943) of *O*. *tsutsugamushi* was propagated in CD-1 Swiss mice at the Naval Medical Research Center, Silver Spring, Maryland, USA [16]. Pre-aliquots of liver-spleen homogenates of *O*. *tsutsugamushi* (Karp strain) at 1x10^6^ murine LD_50_ (MuLD_50_) were prepared and applied to the *O*. *tsutsugamushi* infected macaques as previously described [4, 6]. The control animals received aliquots of liver-spleen homogenates from healthy uninfected mice. A trained veterinarian performed all inoculations on anesthetized macaques via intradermal injection (26G needle) on Inoculation Day 0.

### Experimental design

All macaques were inoculated at the left anterior medial thigh, and were observed daily for clinical signs including the development of skin lesion, regional and generalized lymphadenopathy, loss of appetite, and elevated body temperature. Rectal temperature was measured daily without anesthesia between 15:00 to 15:20 using pole and collar restraint. Local inoculation sites were observed and scored following the Draize and Rise scoring protocols (S1 Table). Blood was drawn every other day for bacterial quantitation (qPCR) and at day 0, 14, and 28 post inoculation (pi) for hematological, biochemical, and immunological bioassays. Control monkeys were euthanized at day 28 pi while *O*. *tsutsugamushi-*infected monkeys were euthanized at day 30 pi, and a panel of tissue specimens for histopathological examination and cerebro-spinal fluid (CSF) were collected for *O*. *tsutsugamushi* qPCR quantification, and immunohistochemical staining.

### Bacterial quantitation

To determine and quantitate the bacterial load of *O*. *tsutsugamushi* post-inoculation, an *O*. *tsutsugamushi*-specific 47kDa gene qPCR assay was performed on blood, CSF, tissues, and eschar swab DNA preparations [6]. The rhesus-specific qPCR assay targeting the single copy macaque oncostatin M (*osm*) gene was used to quantitate host cells in tissue samples as previously described [6, 17]. Results are reported as the ratio of *O*. *tsutsugamushi* to host cell counts. PCR reactions were performed on a CFX96 Real-Time System (BioRad, Foster City, CA, USA), “no template” negative controls were run with each reaction and plasmid DNA served for standard curves in serial dilutions from 10^6^ to 3 copies/μl of 47 kDa protein and macaque *osm* genes. The copy number was calculated from the cycle threshold using Bio-Rad software, and quantitation of 47 kDa protein gene was expressed per 10^4^ macaque cells. The area under the curve was calculated using GraphPad Prism 7 software.

### Hematological and biochemical analyses

Complete blood counts were performed to determine erythrocyte count, hemoglobin, hematocrit, platelet count, leukocyte count, leukocyte differential, mean red blood cell volume, mean red blood cell hemoglobin, mean red blood cell hemoglobin concentration, and mean platelet volume. Plasma collected from heparinized blood was assessed for the concentration of albumin, creatinine, aspartate aminotransferase, alanine aminotransferase, alkaline phosphatase, cholesterol, total bilirubin, urea nitrogen, and creatinine kinase levels.

### Serum levels of circulating soluble cell adhesion molecules (sCAMs)

Soluble E-selectin, L-selectin, ICAM-1, and VCAM-1 in macaque sera were assessed using E-selectin (Abcam, Cambridge, UK), VCAM-1 (Uscn, Wuhan, China), and ICAM-1 (Abnova, Taoyuan, Taiwan) ELISA kits following the manufacturer instructions. Serum samples were diluted 1:10. All samples and standard dilutions were assayed in duplicate, the optical densities measured using a microplate spectrophotometer (Multiskan Go; Thermo scientific, Waltham, MA), and the concentrations of cell adhesion molecules were determined from a standard curve.

### Determination of anti-*O*. *tsutsugamushi* antibody responses (IFA and ELISA)

Serum samples were assessed for *O*. *tsutsugamushi*-specific IgM and IgG antibody titers by indirect immunofluorescence (IFA) and ELISA assays at D0, D14 and D28.

### IFA

Antigen slides coated with *O*. *tsutsugamushi* Gilliam (Burma) and Litchfield (Australia) stains were purchased from the Australian Rickettsial Reference Laboratory (Geelong, Australia). Serum samples were serially diluted two-fold from 1:100 to 1: 25,600 in 2% skimmed milk PBS buffer and incubated onto the antigen slides for 30 min. After washing three times with PBS buffer, slides were incubated with FITC-conjugated goat anti-monkey IgM or IgG (Brookwood Biomedical, Birmingham, AL) for 30 min, and mounted with fluorescence mounting medium (Dako, Glostrup, Denmark) for fluorescence-based microscope endpoint titer determination.

### ELISA

*O*. *tsutsugamushi* strain Karp (Papua New Guinea) whole cell antigen was kindly provided by the Naval Medical Research Center. One-half of a 96-well microtiter plate was coated with *O*. *tsutsugamushi* antigen (100 μl/well), and the other half without antigen (100 μl PBS/well), and stored at 4°C (min. 48 hours). Following steps were performed at room temperature; plates were washed with 0.1% Tween 20 (Sigma, St. Louis, MO, USA) in PBS, and blocked with blocking buffer (5% Skim milk, 0.1% Tween 20 in PBS) for 1 hour. Diluted serum samples (1:100 in blocking buffer), were added to all wells (100 μL/well) and incubated for 1 h. After three wash cycles, incubation with horseradish peroxidase conjugated goat anti-monkey IgG or IgM (Kirkegaard & Perry Laboratories, Gaithersburg, MD) with 100 μl/well at 1:2000 dilution followed for 1 hour. After three wash cycles, ABTS (2,2′-azino-di-(3-ethylbenzthiazoline sulfonic acid)) peroxidase substrate (Kirkegaard & Perry Laboratories, Gaithersburg, MD) was added and incubated for 15 min. Optical densities were measured at 405 nm by Microplate Spectrophotometer (Multiskan Go; Thermo scientific, Waltham, MA), and the net optical density (OD) of each sample was obtained by subtracting the background OD (no antigen reading value) from the *O*. *tsutsugamushi*-antigen OD. Positive serum samples (net OD >0.5) were serially diluted (1:100, 1:400, 1:1600, 1:6400) to determine the endpoint titer. The titers were expressed as the inverse of the highest dilution in which a net OD of ≥ 0.200 was obtained. The mean net OD of three negative control sera was consistently less than an optical density of 0.200.

### Determination of antigen-specific IFN-γ production using ELISpot assay

Peripheral blood mononuclear cells (PBMC) were isolated from 4 ml heparinized blood samples as previous described [18]. ELISpot assays for gamma interferon (IFN-γ) were performed as per the manufacturer’s instruction (Mabtech, 3421M-2A, Stockholm, Sweden) [18]. PBMC were stimulated with 0.2 μg of 47kDa *O*. *tsutsugamushi* antigen (recombinant full-length 47kDa Karp strain was produced and purified by Biomatik, USA). Leucoagglutinin (PHA-L) (Sigma, St. Louis, MO, USA) was used as a positive control at 0.5 μg per well, and there was no antigen in negative control wells.

### Histopathology

After 28 days pi, all infected macaques were euthanized and tissue specimens (normal skin, inoculation site (eschar), draining lymph node, non-draining lymph node, lung, heart, spleen, kidney, liver, bone marrow, mesenteric lymph node, ileum, meninges, brain, brain stem) were collected. All tissues were fixed in 10% neutral buffered formalin solution (Sigma, St. Louis, MO, USA) for at least 2 weeks and processed using an automated tissue processor (SLEE medical, Mainz, Germany). The tissues were then embedded into paraffin blocks and sectioned at 5 μm using a semi-automated rotary microtome (RM2245, Leica, Buffalo Grove, IL, USA). The tissue sections were mounted onto poly L-lysine coated microscope slides, baked at 60 °C for 14–18 h, and stained with hematoxylin-eosin (H&E) or used for immunohistochemistry (IHC). All histopathological sections were evaluated by a board certified veterinary pathologist.

### Immunohistochemistry

The formalin-fixed paraffin embedded tissues were processed as previously described as previously described [6, 19]. The anti-*O*. *tsutsugamushi* monoclonal antibody (clone 1C4B11) of the 56 kDa surface protein of *O*. *tsutsugamushi* was applied at dilution 1:2. Cell marker antibodies used in this study contains CD1a clone NA1/34 at 1:100 (Dako, Glostrup, Denmark), polyclonal CD3 antibody at 1:100 (Dako, Glostrup, Denmark), CD14 clone NCL-L-CD14-223 at 1:75 (Leica, Newcastle, UK), CD31 clone JC70A at 1:2 (OxFab, Oxford, UK), CD68 clone KP-1 at 1:3 (OxFab, Oxford, UK), DCSIGN clone DC28 at 1:200, and HLADR clone CR3/43 at 1:2 (OxFab, Oxford, UK).

Images were acquired and examined using standard fluorescence microscopy (Nikon Eclipse 80i using the NIS element software from Nikon Tokyo, Japan) and a confocal laser scanning microscope (Zeiss LSM 700, using the AxioVision 40 v4.7.1.0 software from Carl Zeiss Imaging Solutions Gmbh, Germany). Images were merged and minimally optimized as a whole file following the requirements for scientific imagery [20], using Photoshop CS3 extended, version 10.0.

### Statistical analyses

Statistical analyses were performed using GraphPad Prism Software v. 7 or STATA version 14 SE (StataCorp, Texas, USA). The results between the infected and non-infected groups were compared using the non-parametric Mann-Whitney *U-* test. The data of surrogate markers were expressed as median and inter-quartile range, unless otherwise stated. Significant differences between time points within a group were determined with the non-parametric Wilcoxon t-test. Two-tailed P values less than 0.05 were considered significant.

## Results

### Clinical signs and symptoms

**Fig 1:**
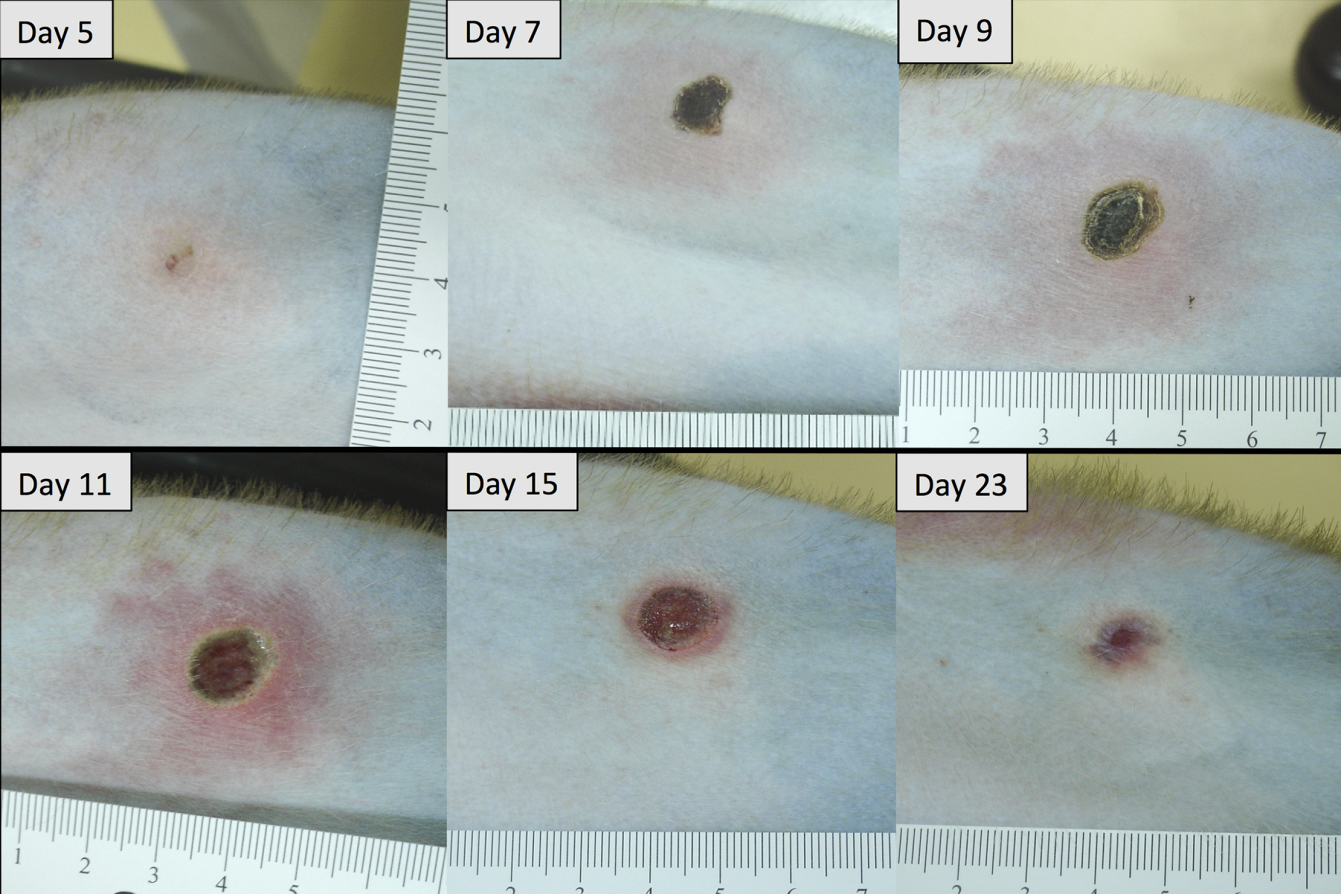
Eschar development at the inoculation site of rhesus macaques (BR1-03) inoculated with *O*. *tsutsugamushi* Karp strain.

**Fig 2:**
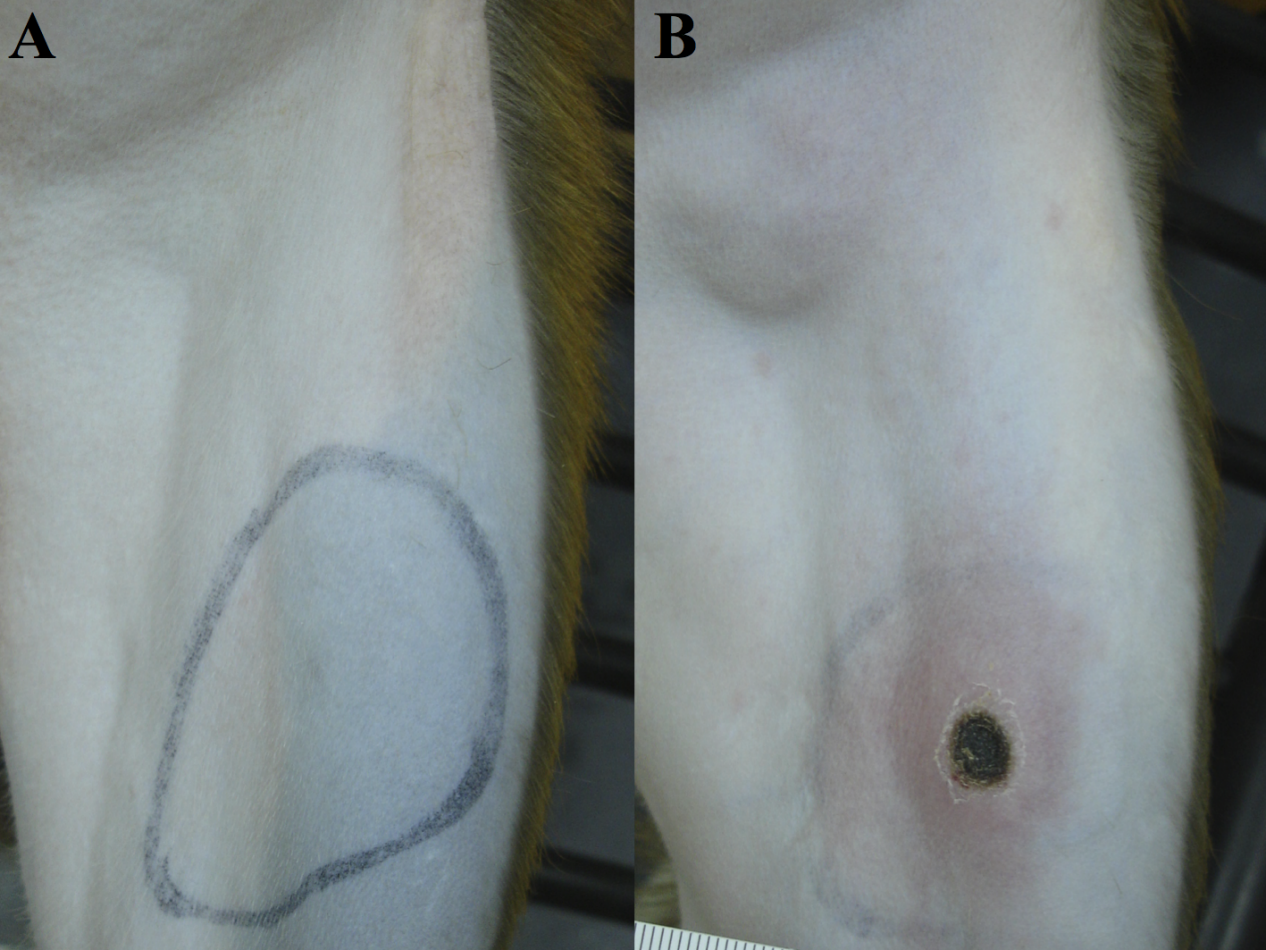
Regional lymphadenopathy and eschar formation of inoculated rhesus macaques on day 10 pi. (A) control macaque (BR1-04), (B) *O*. *tsutsugamushi* inoculated macaque (BR1-02).

**Fig 3:**
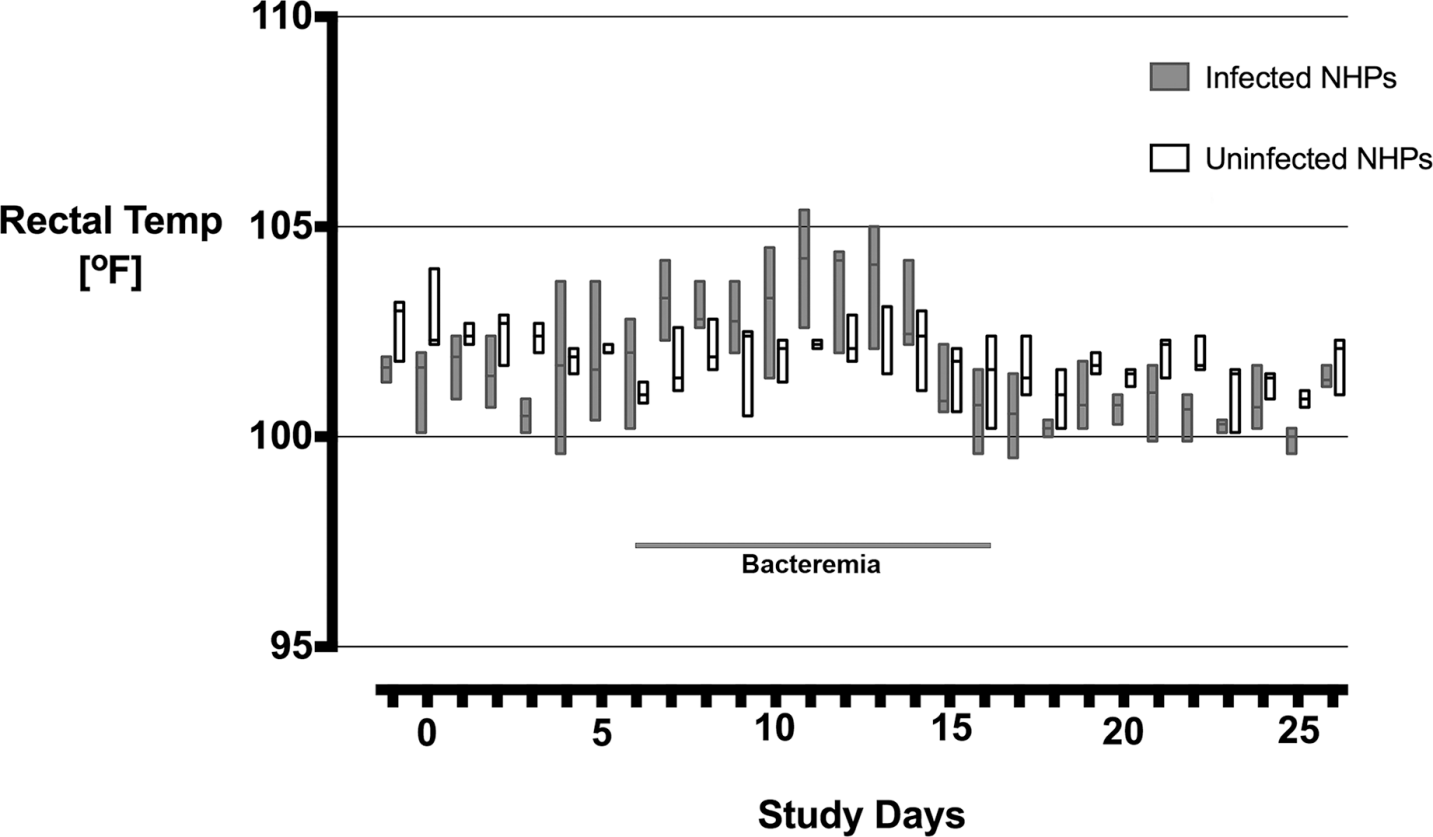
Rectal temperature of *O*. *tsutsugamushi* inoculated rhesus macaques. The distribution of median (range) of rectal temperatures compared between inoculated and uninfected macaques, was significantly raised in the infected macaque group during the time of bacteremia from day 6 to day 16, (Mann Whitney U test, p = 0.033).

All *O*. *tsutsugamushi* inoculated macaques developed classical eschar lesions at the injection site within 7 days. The eschars presented as small indurated, erythematous, vesiculo-papules on day 5, with moderate perifocal erythema and edema, which grew in diameter with increasing erythema, perifocal edema and demarked excoriation resulting in a dark central necrosis with black crust and a raised indurated border on day 7. Eschars were completely painless, with a round shape, indurated border and a necrotic zone of 6–10 mm in diameter. They reached maximum size on day 10–12, and then decreased slowly in size until day 21–23 to leave a hyperpigmented area, but no scar (Fig 1). Explicit regional lymphadenopathy (inguinal draining lymph node) was observed in all *O*. *tsutsugamushi* inoculated macaques starting on day 7 which developed into generalized lymphadenopathy and resolved before day 21 (Fig 2). During the bacteremia phase (median qPCR-positivity from Day 6 to Day 16) the infected macaques demonstrated a significantly higher core temperature (p = 0.0335), when the distribution of the median rectal temperatures was compared. The duration of fever was 7 days (Fig 3), and interestingly, a brief paradoxical drop in rectal temperature was observed before the onset bacteremia (as noted previously in cynomolgus macaques) [4].

### Hematology and biochemistry

On day 14 pi, hematological analyses demonstrated significantly elevated leukocyte (WBC) counts in *O*. *tsutsugamushi* inoculated macaques compared to control macaques, and no differences in erythrocyte (RBC) counts, hematocrit (Hct), hemoglobin (Hb), platelet, mean red blood cell volume (MCV), mean red blood cell hemoglobin (MCH), and mean red blood cell hemoglobin concentration (MCHC) (S2 Table). Biochemical analyses demonstrated significant changes of albumin and liver enzyme levels (aspartate transaminase, alanine transaminase, and alkaline phosphatase), but not creatinine, cholesterol, total bilirubin, urea nitrogen, and creatinine kinase levels (S3 Table).

### Bacteremia

To detect bacteremia in macaques, whole blood samples were collected every two days for DNA extraction and 47 kDa *O*. *tsutsugamushi* qPCR assay. *O*. *tsutsugamushi* DNA was first detected on day 6 for all inoculated macaques. The duration of bacteremia ranged from 6 to 18 days, and the bacterial loads ranged from 1 to 85 organisms / 10,000 macaque cells per timepoint (Table 1). Additional eschar swab specimens on days 12 and 14 demonstrated the presence of *O*. *tsutsugamushi* DNA in all samples. To reduce eschar crust damage only 2 swab samples were performed. A large bacterial load was detected in animal BR1-02’s eschar, which correlated with bacteremia results (Table 1).

**Table 1:** Summary of clinical observations of *O*. *tsutsugamushi* inoculated rhesus macaques.

Subject ID	Eschar	Draining LN	Bacteremia	Area under the bacteremia curve
BR1-01	Day 6–16	Lymphadenopathy	Day 6–16	10
BR1-02	Day 6–16	Lymphadenopathy	Day 6–16	182
BR1-03	Day 6–18	Lymphadenopathy	Day 6–18	16
BR1-05	Day 6–12	Lymphadenopathy	Day 6–12	16

### Circulating soluble cell adhesion molecules

**Fig 4:**
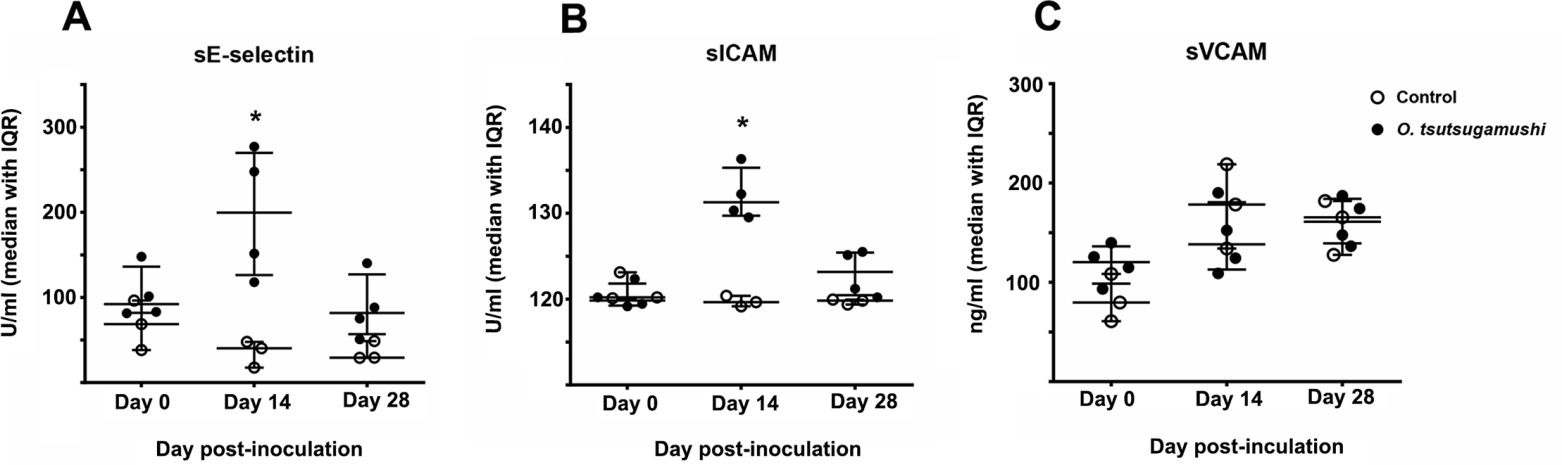
**Time course of serum levels of soluble cell adhesion molecules in macaques, (A) sE-selectin, (B) sICAM, (C) sVCAM.** Dot plots demonstrating the serum levels of soluble cell adhesion molecules of infected (n = 4, solid circles) and controls (n = 3, hollow circles) macaques. Bars indicate median and interquartile ranges (IQR). The asterisk indicates significant difference (P<0.05) when compared between *O*. *tsutsugamushi*-infected and control group results.

Evidence for endothelial and leukocyte activation was found by increased serum levels of soluble cell adhesion molecules (sE-selectin, sICAM-1, and sVCAM-1) in *O*. *tsutsugamushi*-infected macaques, when compared to mock-infected macaques. Soluble E-selectin and sICAM-1, but not VCAM-1 serum levels were significantly elevated on day 14 pi compared to controls (Fig 4).

### Anti-*O*. *tsutsugamushi* antibody response

**Fig 5:**
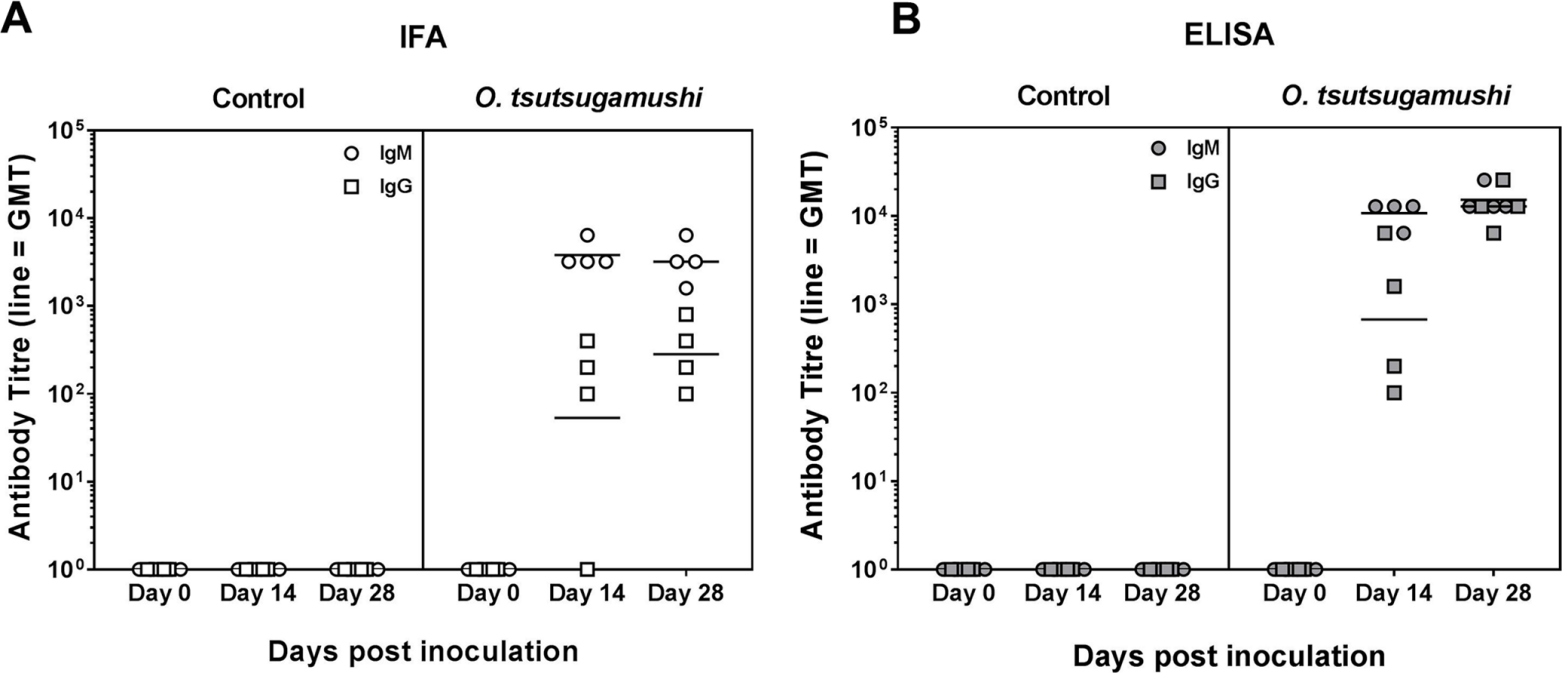
**Scrub typhus specific antibody titers in serum of *O*. *tsutsugamushi* inoculated macaques after inoculation, (A) IFA and (B) ELISA.** Data are shown as antibody titers for IFA and net OD translated into titers of IgM and IgG antibodies in infected macaques (n = 4). Bars indicate the geometric mean.

The IgM antibody titers increased rapidly to reach their highest dilution before D14 in both IFA and ELISAs, while IgG levels increased more slowly and were highest at the D28 timepoint. The results from ELISA shown higher antibody titers compared to IFA results (Fig 5). IgM and IgG antibody titers against *O*. *tsutsugamushi* increased in all infected macaques (n = 4) and remained undetectable in the control group (n = 3).

### Determination of antigen-specific IFN-γ production using ELISpot assay

**Fig 6:**
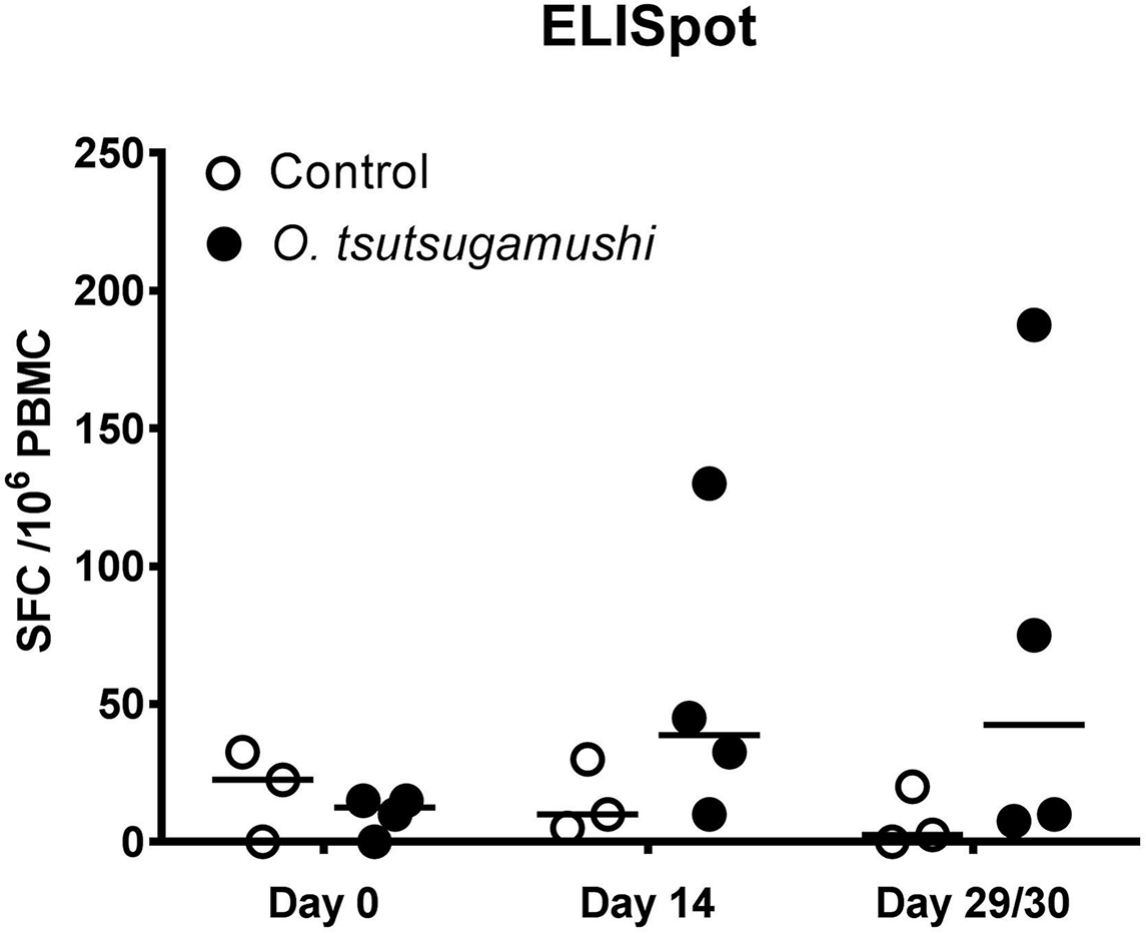
*Ex vivo* production of antigen-specific IFN-γ by PBMC of inoculated macaques. Data are shown as spot forming cells per million peripheral blood mononuclear cells (SFC /10^6^ PBMC) of individual macaques (Control; n = 3, *O*. *tsutsugamushi* inoculation; n = 4), following 18 hours of stimulation with r47 protein. Bars indicated median.

The isolated PBMCs were subjected to the *O*. *tsutsugamushi* antigen-specific IFN-γ production ELISpot assay. The median IFN-γ production increased in *O*. *tsutsugamushi* inoculated macaques over time, and although the range was wide all *O*. *tsutsugamushi* inoculated macaques showed induction of IFN-γ at D28 (Fig 6).

### Histopathology

The histopathological evaluation of the submitted tissues noted no significant pathological findings in 16 tissues of *O*. *tsutsugamushi* inoculated macaques when compared with control macaques at D29, suggestive of complete recovery.

### Detection of *O*. *tsutsugamushi* in macaque tissues at D28

*O*. *tsutsugamushi* DNA was detected by qPCR in eschar and spleen samples of macaque BR1-02, the draining lymph node of BR1-03, and in the non-draining lymph node in BR1-05. The eschar sample contained the highest number of *O*. *tsutsugamushi* organisms compared to other tissues.

Immunohistochemistry staining of these tissue specimens with anti-*O*. *tsutsugamushi* monoclonal antibody 1C4B11 demonstrated *O*. *tsutsugamushi* organisms in the dermis of the eschar and in the parenchyma of all three tissues, and a large number of *O*. *tsutsugamushi* organisms was observed in the eschar compared to spleen, draining lymph node, and non-draining lymph node.

### Phenotyping of *O*. *tsutsugamushi* infected cells

**Fig 7:**
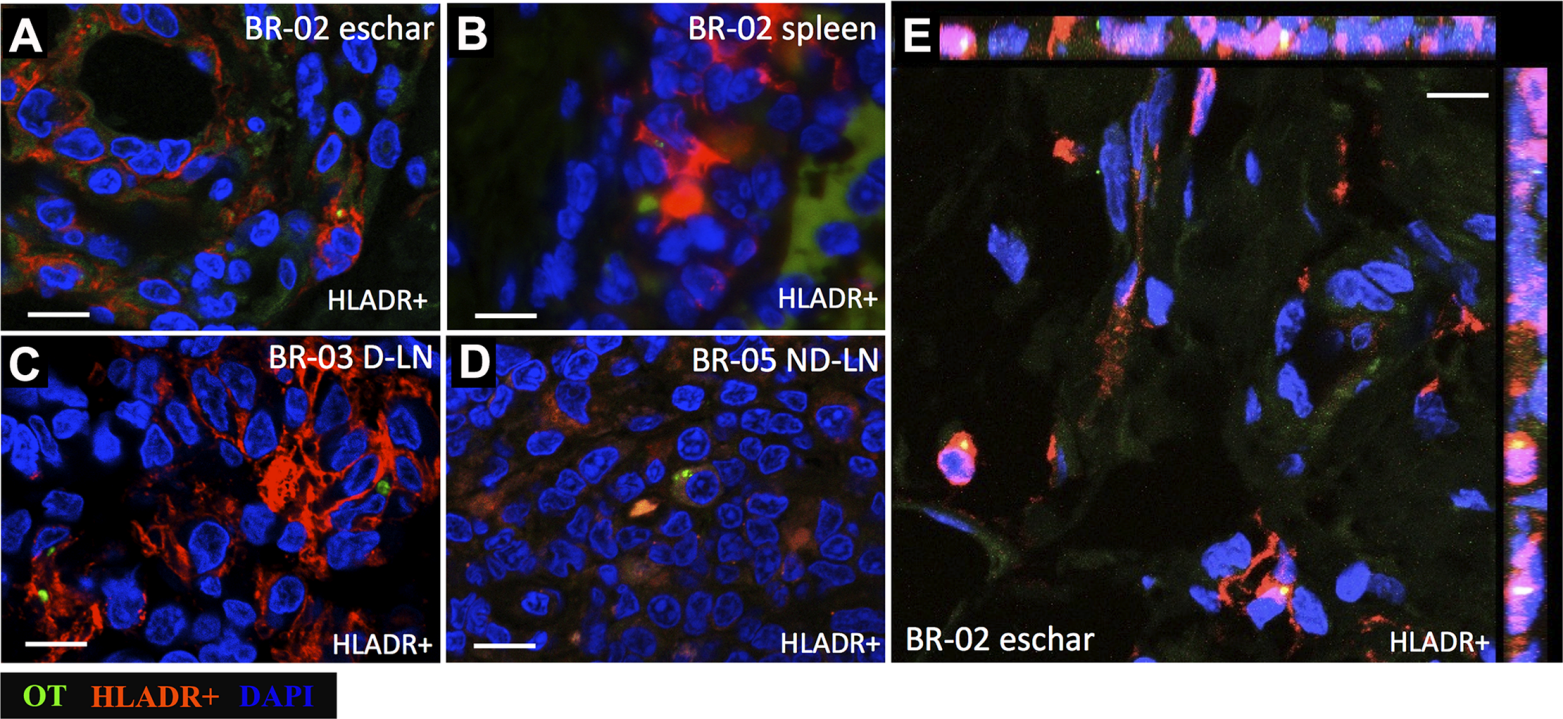
**Localization of intracellular *O*. *tsutsugamushi* in antigen presenting cells (APCs) in macaque tissues, (A and E) Eschar: BR1-02, (B) Spleen: BR1-02, (C) Draining lymph node (D-LN): BR1-03, (D) Non-draining lymph node (ND-LN): BR1-05 after 28 day pi.** *O*. *tsutsugamushi* is labeled in green, APCs are characterized by MHC class II receptor (HLADR) and shown in red. Panels A-D show *O*. *tsutsugamushi* organisms associated with APCs in eschar, spleen, draining and non-draining lymph nodes. Panel E shows orthogonal views of confocal Z-stack images. DAPI nuclear counterstain in blue. Scale bars: 10 μm.

**Fig 8:**
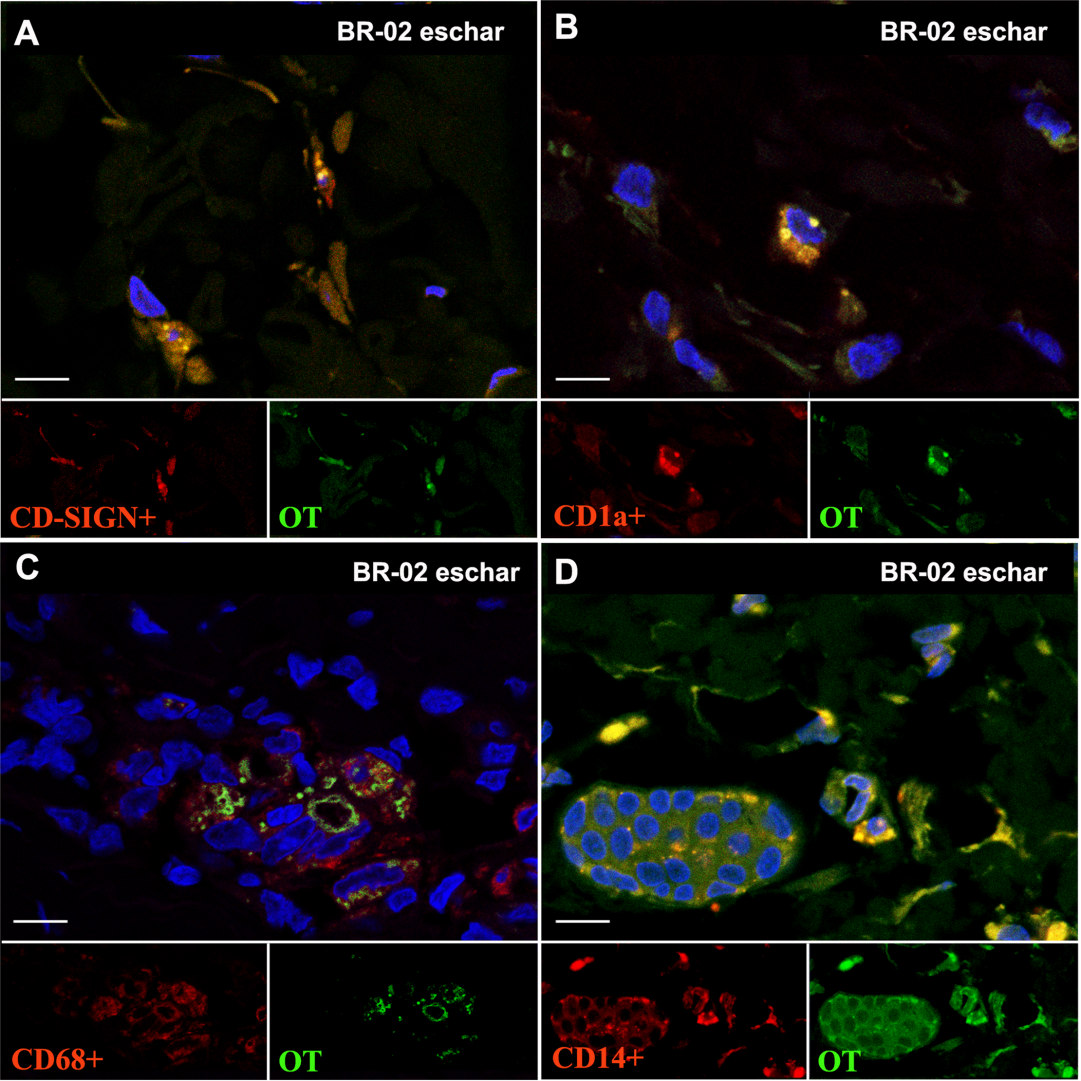
**Co-localization of intracellular *O*. *tsutsugamushi* at macaque immune cells;** (A) Dendritic cells; DC-SIGN labeling (B) Dendritic cells; CD1a labeling, (C) Macrophages; CD68 labeling, (D) Monocytes/macrophages; CD14 labeling after 28 days pi. *O*. *tsutsugamushi* is labeled in green, and immune cells are shown in red. DAPI nuclear counterstain in blue. Split green/red channel sub-images and the overlay in the larger panel. Scale bars: 10 μm.

The highest number of intracellular *O*. *tsutsugamushi* organisms was observed in antigen-presenting cells (APCs, HLADR+, Fig 7). In the eschar (BR1-02), *O*. *tsutsugamushi* was associated with dendritic cells (HLADR+, DCSIGN+, CD1a+) and monocyte/macrophage (CD68+, CD14+), but not T cells (CD3+) and endothelial cells (CD31+) (Fig 8), while in the spleen section of the same macaque the only association of *O*. *tsutsugamushi* with a leucocyte phenotype was within APCs (HLADR+) (Fig 7). For the draining and non-draining lymph node samples, *O*. *tsutsugamushi* was found to associate with APCs (HLADR+) (Fig 7).

## Discussion

### Scrub typhus in rhesus macaques closely resembles human disease

This study characterized a rhesus macaque intradermal inoculation model of scrub typhus in view of future vaccine development studies. Human scrub typhus is characterized by a febrile illness, often with nonspecific symptoms, an early bacteremia period, during which an inoculation eschar can develop, possible skin rash, a strong association with raised liver transaminases and regional lymphadenopathy after ID inoculation of *O*. *tsutsugamushi* via mite bite, and subsequent establishment of humoral and cellular immune responses [12, 21–24]. The clinico-pathophysiological responses observed in rhesus macaques after ID *O*. *tsutsugamushi* inoculation demonstrated striking similarity to human disease, with development of fever during bacteremia, marked eschar formation, regional followed by generalized lymphadenopathy, similar bacteremia onset and duration, altered liver function, increased WBC counts, and pathogen-specific antibody (IgM and IgG) and cell-mediated immune responses. Additionally, time course sCAM serum levels demonstrated endothelial and leucocyte activation in analogy to published observations in humans [25].

Although pathophysiological features of human scrub typhus have been described in a cynomolgus macaque (*Macaca fascicularis*) ID inoculation model, these data have not been challenged by or compared to any alternative models i.e. rhesus macaques [26]. The rhesus macaque response was characterized with an earlier onset of clinical disease, a more pronounced eschar formation, stronger liver function damage and a bacteremia phase that corresponded more closely with observations in humans (D6-16) (Table 2). Importantly, the features and lesions serving as phenotypic correlates of immune protection described in our recent scrub typhus vaccine-challenge study in cynomolgus macaques [3, 4, 13], were found to be more pronounced and resembled more closely to human disease dynamics in the rhesus macaque (*Macaca mulatta*) model: the distribution of fever was significantly raised during the entire bacteremia phase; the eschar lesions were larger in size, more clearly delineated and invariably demonstrated induration, erythema and central necrosis–features required for the RISE score (S1 Table); bacteremia curves were consistent in onset (time-to-bacteremia); and biochemistry/hematological markers (albumin reduction, increased liver transaminases and leukocytosis) were more pronounced than previously in the cynomolgus macaque model.

**Table 2:** Comparison of clinical scrub typhus characteristics between cynomolgus and rhesus macaques.

Markers and clinical signs	Cynomolgus macaques	Rhesus macaques (*this study*)	Human scrub typhus	References
**Fever**	Onset day 10	Onset day 7	Onset day 8	[4, 12, 30]
	Peaked on day 14	Peaked on day 11	Peaked on day 11	
	Duration days 12–19 (7 days)	Duration days 7–14 (7 days)	Duration NA (due to treatment or incomplete data)*	
**Eschar**	Onset day 9	Onset day 6	Onset day 5–7	[4, 19, 30]
	Peaked on day 15	Peaked on day 9	Peaked on day 9	
	Duration days 10	Duration days 12	Duration NA (due to treatment or incomplete data)	
	*Heterogeneous eschar manifestation*, *moderate severity*, *not all macaques developed eschars*.	*All animals developed full blown eschars with necrotic centers*, *uniformly severe eschar formation*.	*All inoculated naïve volunteers developed full blown eschars with necrotic centers*.	
**Bacteremia**	Onset day 9 (mean day 10)	Onset day 6 (mean day 6)	Onset day 8 (mean day 9)	[4, 30, 31]
	Peaked on day 16	Peaked on day 11	Peaked on days 8–10	
	Duration days 9–21 (11 days)	Duration days 6–16 (10 days)	Duration days NA (treated)	
**Lymphadenopathy**	Onset 3–4 days before fever onset (all animals with LA)	Onset 1–2 days before fever onset (all animals with LA)	NA. LA onset usually coinciding with fever onset (approx. 60% of patients)	[4, 30]
**Hematology and Biochemistry**	Significant differences in levels of platelets, leukocyte and lymphocyte counts, protein, albumin, globulin, albumin/globulin ratio, alkaline phosphatase, urea nitrogen, and creatinine kinase (no changes in LFTs)	Significant differences in levels of leukocyte counts, albumin, alkaline phosphatase, LFTs	Significant differences in levels of platelets, leukocyte and lymphocyte counts, protein, albumin, alkaline phosphatase, LFTs, urea nitrogen, creatinine kinase, C-reactive protein, LDH	[4, 30, 32]
**Cell adhesion molecules**	NA	Elevated serum levels of soluble cell adhesion molecules: sE-selectin, sICAM-1, and sVCAM-1	Elevated sL-selectin levels correlated with skin rash duration, eschar, elevated WBCs, lymphocytes, and neutrophils	[25, 32]
		(correlations NA)	Elevated sE-selectin levels correlated with illness duration, lymphadenopathy, eschar, elevated WBCs and neutrophils	
**Cellular tropism**	Immunophenotyping of infected cells included CD3, HLA-DR, CD68 and neutrophil lysozyme	Immunophenotyping of infected cells included CD3, CD4, CD8, HLA-DR, CD68, CD14, DC-SIGN, CD1a, CD20, CD15 and lysozyme	see ref [19]	[4, 19]
**Additional considerations**	Limitations in availability and compatibility of reagents and antibodies.	Extensive characterization through HIV/SIV studies, wide range of reagents, detailed immunological data available	Only limited time course data available on relevant markers and clinical signs to date.	
	Immune response dynamics comparable to humans for IgG, IgM, CMI (crude OT-WCA), ref [4]	Immune response dynamics comparable to humans for IgG, IgM, CMI (optimised OT-WCA, recombinant p47kDa), ref [18]	Immune are incompletely characterized highly limited antigen-specific, time course or longitudinal data (IgG, IgM, CMI (optimized WCA-OT), ref [18]	
	Most closely related model: scrub typhus (*O*. *tsutsugamushi*)	Most closely related model: Rocky mountain spotted fever (*Rickettsia rickettsii*)		

Note: NA not available; Days are post inoculation (pi), except where stated; LFT liver function tests (AST, ALT); OT-WCA *O*. *tsutsugamushi* whole cell antigen.

* One study in chigger-challenged healthy volunteers were exposed to chiggers for 9 days and as such could not be included for time course data [23].

The rhesus macaque is among the best-known species of Old World monkeys, and due to its wide distribution, and large population, it is listed as “least concern” in the IUCN Red List of Threatened Species. Although rhesus macaques have been well studied for a number of important infectious diseases like tuberculosis, malaria, HIV/SIV-AIDS and recently for Zika, it is important to remain cautious when interpreting data generated in rhesus macaque models in the absence of known human disease mechanisms, as these may differ and prove imperfect for research in certain conditions [27–29]. The extent of research involving rhesus macaques and the broad availability of validated laboratory reagents, especially leucocyte immune-phenotyping reagents (i.e. mAbs, ELISAs, histopathology reagents, cytokines etc.) as well as published procedures and protocols (i.e. antigen retrieval, antibody dilution optimizations and conditions etc.) currently allow for more in-depth investigations in rhesus than cynomolgus laboratory NHP models.

The available data on human scrub typhus was summarized for comparative purposes in this study (Table 2), and is a non-exhaustive collation from non-standardized investigations and serves for approximate guidance; these data were retrieved from old publications, where scrub typhus pre-exposed and naïve volunteers were ID inoculated using *O*. *tsutsugamushi* suspensions. Data on biochemistry, hematology and bacteremia included recent reports from clinical patient series from Laos and Thailand.

### Bacteremia, fever and symptoms onset post-inoculation

Our previous study with cynomolgus macaques used the same inoculum preparation (mouse liver/spleen homogenate, dosed at 10^6^ muLD_50_) as this study, but the bacteremia period in rhesus macaques was shorter ranging from D6-D16 post-inoculation, than in the cynomolgus macaques (ranged from D9-D21). The shorter duration and earlier onset of bacteremia is much closer to human findings, where *O*. *tsutsugamushi* qPCR positivity is found as early as D2 up to D12 of fever manifestation (not post-inoculation) [33]. Early studies involving human volunteers have demonstrated that a latency of approximately 3–4 days following the mite inoculation before the onset of fever [23]. Hence, the human “rickettsemia phase” can be estimated at approximately D5-6 to D15 post-inoculation, which is similar to our findings in rhesus model.

A delay between onset of symptoms after pathogen exposure could be associated with pre-existing partially protective immunity (disease endemic regions), variation of inoculum dosage, strain virulence and the inoculum route applied [3, 4, 12, 23]. Further characterization of the effects of these factors on disease onset and manifestation is crucial, as they relate to the major phenotypic correlates of immune protection in scrub typhus (fever, bacteremia, inoculation lesion characteristics) and should be an object of future investigations [4]. The rhesus macaques developed significantly raised core temperatures during the bacteremia phase–a clinically highly relevant feature used to estimate the bacteremia phase in humans. An animal model that does not develop fever will always be limited due to its dependence on bacterial dissemination dynamics–a feature not regularly assessed in humans. The transformation of fever time course data into area under the curve for comparisons between interventional/vaccine groups are important, as these data correlated previously with the density of bacteremia [4, 33].

### Cell adhesion molecules and cellular tropism of *Orientia tsutsugamushi*

The sCAM time course profiles with significant rise in sE-selectin and sICAM plasma levels (unfortunately no rhesus sL-selectin assays were available at this time) provide additional support to corroborate the usefulness of this model for scrub typhus; a previous study in humans demonstrated that elevated sE-selectin levels correlated significantly with the duration of fever/illness before admission, the presence of eschar formation and lymphadenopathy, as well as elevated WBCs and neutrophils [25]. The sample size of this study did not allow for correlations to be made, but we anticipate these markers to be useful in future evaluations.

Immunophenotyping of infected host leucocytes revealed intracellular *O*. *tsutsugamushi* infection only within host monocytes and dendritic cells in the skin (inoculation site), spleen, draining and non-draining lymph node specimens, representing identical findings of the cellular tropism for *O*. *tsutsugamushi* in humans [19]. *O*. *tsutsugamushi* infected exclusively APCs—also in agreement with previous findings in human eschar biopsies—and no evidence of endothelial infection was seen, although the co-localization imaging was done on D28 post-mortem samples. The availability of validated antibodies for rhesus enabled characterization of infected leucocyte subsets, including "inflammatory" monocytes expressing CD14/CD68, dendritic cell phenotypes with CD1a/DCSIGN positivity, but no intracellular infection of T-cells, or endothelial cells was found. The new finding of numerous intact *O*. *tsutsugamushi* within Langerhans cells, monocytes and dermal dendritic cells at the inoculation site following resolution of infection, is suggestive of ongoing replication in the skin throughout the disease course, even after healing of the eschar lesion (in untreated cases). The absence of endothelial infection and scarring/sequelae dynamics again compare well with findings in human eschar biopsies [19].

At termination of the study (D28), the tissue specimens containing *O*. *tsutsugamushi* DNA, as determined by qPCR-assay, included skin (inoculation site), spleen, draining and non-draining lymph node specimens, suggesting that either these tissues had particularly high bacterial loads, or that they might be associated with bacterial persistence and potential relapse in case of inadequate treatment [34]. All infected host cells in lymph nodes and spleen at D28 were APCs–these cellular subsets may represent a niche for this obligate intracellular bacterium, and pathogen-host immunomodulation is likely to occur within them. No viability testing was performed in this study, and no evidence for *O*. *tsutsugamushi* was found in lung and/or liver as recently described in persistent infections in a C57BL/6 mouse model [35, 36]. Additional investigations into the phenotypes of infected cells in lymph node and other organs throughout the disease course need to precisely address where orientiae invade, survive and replicate, as this will contribute to our understanding of the immunopathophysiology and facilitate discovery of immune correlates of protection in scrub typhus.

### Immune responses

In humans, natural protective immunity against *O*. *tsutsugamushi* requires both humoral and cell-mediated responses [21, 22, 37]. The antibody response dynamics in rhesus macaques were identical to those observed previously in cynomolgus macaques, with increasing titers at D14 and all animals reaching maximum titers at D28 for both IgG and IgM. Cell-mediated immune responses (CMI) were seen in all infected macaques at D28 of the study, however only 2 macaques demonstrated strong responses (>500SFC/mio PBMC). In subsequent studies we have further optimized antigen and ELISpot assay conditions and observed consistent CMI responses [18]. These results are comparable to responses seen in the control macaque group in the previous cynomolgus study, where unvaccinated controls did not exhibit an increase in the number of antigen-specific IFN-γ secreting cells until D21 after ID challenge with *O*. *tsutsugamushi* [4].

The 47kDa *htrA* antigen ELISpot assay used in this study (based on peptide pools) was chosen due to promising findings with a 47kDa *htrA* based vaccine candidate in a previous study [4]. The cell-mediated immune response induced by the 47kDa *htrA* gene co-presented with the pRhGM-CSF plasmid adjuvant was more rapid than the 47kDa antigen-specific cellular response of the natural immune response. This may also be associated with the inoculum route (intradermal) or inoculum dose, where a smaller dose would result in a delayed bacteremia and followed by a delayed cell-mediated response. The establishment of the strong cell-mediated immune response in rhesus macaques (measured by a 47kDa *htrA* peptide-pool), is further evidence for the suitability in vaccine evaluations, as IFN-γ and type-1 immune responses have been associated with protection from *O*. *tsutsugamushi* infections in animal models, and strong IFN-γ responses to *O*. *tsutsugamushi* infection have been associated with acute scrub typhus in humans [3, 4, 12, 13, 21, 22, 38].

In summary, when comparing findings between both macaque models using the same inoculation preparation, strength and route, the rhesus macaques unequivocally produced more reliable classic eschar formation with central necrotic crusts at the inoculation site than the cynomolgus model. This study characterized scrub typhus disease features in rhesus macaques, and provided data on cell adhesion molecule dynamics and the cellular tropism of *O*. *tsutsugamushi* in rhesus macaques, which were similar to findings described in humans and of high relevance in evaluating new animal models for vaccine development.

Importantly, the time course dynamics of eschar formation, temperature and bacteremia–which all represent phenotypic correlates of immune protection in scrub typhus—occurred earlier and more pronounced in the rhesus model. The aim was not to develop a lethal model, but compare previous findings in cynomolgus macaques to those in rhesus macaques. Taking into account all findings, the rhesus macaques disease characteristics following ID inoculation resembled that of human patients with scrub typhus more closely and importantly more consistently than cynomolgus macaques. In addition, the access to a broader panel of validated immunologic assays/reagents for the rhesus macaques compared to cynomolgus and other NHPs makes this laboratory animal a more useful model for scrub typhus to investigate immunopathology, correlates of immune protection and vaccine candidate evaluations.

Further characterization and development of the rhesus macaque model for scrub typhus should include the effect of increasing inoculum dosages, the variation of immunopathophysiological responses and cross-protection to *O*. *tsutsugamushi* strains, treatment studies, and assessment of elicited immune response dynamics of immunogenic antigens. These data will enable more in-depth evaluation of correlates of protection for both natural and vaccine induced immunity, to subsequently support the evaluation of future vaccine candidates against scrub typhus.

## Supporting information

S1 Table Draize and RISE scores of control and *O*. *tsutsugamushi*-infected macaques. RISE score (1–3) = R = Redness erythema, I = Induration of the skin, S = Swelling/edema of skin, E = Eschar formation.
(DOCX)

S2 Table Hematological values of control and *O*. *tsutsugamushi*-infected macaques. Red blood cell (RBC), hematocrit (Hct), hemoglobin (Hb), white blood cell (WBC), mean red blood cell volume (MCV), mean red blood cell hemoglobin (MCH), and mean red blood cell hemoglobin concentration (MCHC).
(DOCX)

S3 Table Biochemical values of control and *O*. *tsutsugamushi*-infected macaques. Albumin (ALB), alkaline phosphatase (ALP), alanine transaminase (ALT), aspartate transaminase (AST), liver enzyme levels, were significantly raised at day 14, when compared to control macaques—but not creatinine, cholesterol, total bilirubin (TBIL), urea nitrogen, and creatinine kinase (CPK) levels.
(DOCX)
