# Supplementary material for: Characterization of the rhesus macaque (Macaca mulatta) scrub typhus model: Susceptibility to intradermal challenge with the human pathogen Orientia tsutsugamushi Karp
Source: PLoS Negl Trop Dis. 2018 Mar 9;12(3):e0006305. doi: 10.1371/journal.pntd.0006305 (PMC5862536; doi:10.1371/journal.pntd.0006305)
Supplement: S1 Table — RISE score (1–3) = R = Redness erythema, I = Induration of the skin, S = Swelling/edema of skin, E = Eschar formation. (DOCX) [file pntd.0006305.s001.docx]

**Table S1. Draize and RISE scores of control and *O. tsutsugamushi*-infected macaques.**RISE score(1-3) = R = Redness erythema, I = Induration of the skin, S = Swelling/edema of skin, E = Eschar formation.

| **ID** | **Infection** | **Day 0** | | | | | | | **Day 5** | | | | | | | **Day 7** | | | | | | | **Day 9** | | | | | | |
| --- | --- | --- | --- | --- | --- | --- | --- | --- | --- | --- | --- | --- | --- | --- | --- | --- | --- | --- | --- | --- | --- | --- | --- | --- | --- | --- | --- | --- | --- |
|  |  | **Score** | | | | **Diameters (mm)** | | **Draize**  **Score** | **Score** | | | | **Diameters (mm)** | | **Draize**  **Score** | **Score** | | | | **Diameters (mm)** | | **Draize**  **Score** | **Score** | | | | **Diameters (mm)** | | **Draize**  **Score** |
|  |  | **R** | **I** | **S** | **E** | **R** | **E** |  | **R** | **I** | **S** | **E** | **R** | **E** |  | **R** | **I** | **S** | **E** | **R** | **E** |  | **R** | **I** | **S** | **E** | **R** | **E** |  |
| BR1-01 | Ot-infected | 0 | 0 | 0 | 0 | 0 | 0 | 0 | 2 | 0 | 3 | 0 | 10 | 15 | 2 | 3 | 0 | 3 | 3 | 35 | 25 | 4 | 3 | 0 | 3 | 3 | 45 | 20 | 4 |
| BR1-02 | Ot-infected | 0 | 0 | 0 | 0 | 0 | 0 | 0 | 2 | 0 | 3 | 0 | 6 | 11 | 2 | 1 | 0 | 0 | 1 | 20 | 0 | 3 | 2 | 0 | 3 | 2 | 27 | 17 | 3 |
| BR1-03 | Ot-infected | 0 | 0 | 0 | 0 | 0 | 0 | 0 | 3 | 0 | 2 | 0 | 12 | 8 | 2 | 1 | 0 | 0 | 2 | 25 | 0 | 3 | 2 | 0 | 3 | 2 | 32 | 20 | 3 |
| BR1-05 | Ot-infected | 0 | 0 | 0 | 0 | 0 | 0 | 0 | 3 | 0 | 3 | 2 | 15 | 10 | 3 | 3 | 0 | 3 | 3 | 28 | 15 | 4 | 3 | 0 | 3 | 3 | 33 | 20 | 4 |
| BR1-04 | Control | 0 | 0 | 0 | 0 | 0 | 0 | 0 | 0 | 1 | 0 | 0 | 0 | 0 | 1 | 0 | 0 | 0 | 0 | 0 | 0 | 0 | 0 | 0 | 0 | 0 | 0 | 0 | 0 |
| BR1-06 | Control | 0 | 0 | 0 | 0 | 0 | 0 | 0 | 1 | 0 | 0 | 0 | 7 | 0 | 1 | 0 | 1 | 0 | 0 | 0 | 0 | 0 | 0 | 0 | 0 | 0 | 0 | 0 | 0 |
| BR1-07 | Control | 0 | 0 | 0 | 0 | 0 | 0 | 0 | 1 | 1 | 0 | 0 | 5 | 0 | 1 | 0 | 1 | 0 | 0 | 0 | 0 | 0 | 0 | 0 | 0 | 0 | 0 | 0 | 0 |

| **ID** | **Infection** | **Day 11** | | | | | | | **Day 15** | | | | | | | **Day 23** | | | | | | |
| --- | --- | --- | --- | --- | --- | --- | --- | --- | --- | --- | --- | --- | --- | --- | --- | --- | --- | --- | --- | --- | --- | --- |
|  |  | **Score** | | | | **Diameters (mm)** | | **Draize**  **Score** | **Score** | | | | **Diameters (mm)** | | **Draize**  **Score** | **Score** | | | | **Diameters (mm)** | | **Draize**  **Score** |
|  |  | **R** | **I** | **S** | **E** | **R** | **E** |  | **R** | **I** | **S** | **E** | **R** | **E** |  | **R** | **I** | **S** | **E** | **R** | **E** |  |
| BR1-01 | Ot-infected | 3 | 0 | 3 | 3 | 35 | 20 | 4 | 1 | 3 | 0 | 3 | 20 | 0 | 4 | 2 | 2 | 0 | 2 | 10 | 0 | 2 |
| BR1-02 | Ot-infected | 3 | 0 | 3 | 2 | 35 | 25 | 3 | 2 | 3 | 0 | 3 | 18 | 0 | 4 | 2 | 2 | 0 | 2 | 10 | 0 | 3 |
| BR1-03 | Ot-infected | 3 | 0 | 3 | 3 | 30 | 18 | 4 | 2 | 3 | 0 | 3 | 15 | 0 | 4 | 2 | 2 | 0 | 2 | 9 | 0 | 2 |
| BR1-05 | Ot-infected | 3 | 0 | 3 | 2 | 25 | 18 | 3 | 2 | 3 | 0 | 2 | 14 | 0 | 3 | 2 | 2 | 0 | 2 | 8 | 0 | 2 |
| BR1-04 | Control | 0 | 0 | 0 | 0 | 0 | 0 | 0 | 0 | 0 | 0 | 0 | 0 | 0 | 0 | 0 | 0 | 0 | 0 | 0 | 0 | 0 |
| BR1-06 | Control | 0 | 0 | 0 | 0 | 0 | 0 | 0 | 0 | 0 | 0 | 0 | 0 | 0 | 0 | 0 | 0 | 0 | 0 | 0 | 0 | 0 |
| BR1-07 | Control | 0 | 0 | 0 | 0 | 0 | 0 | 0 | 0 | 0 | 0 | 0 | 0 | 0 | 0 | 0 | 0 | 0 | 0 | 0 | 0 | 0 |

**Reaction Codes:**

**RISE score =**

**R =** Redness erythema, **I =** Induration of the skin (=infiltration), **S =** Swelling/edema of skin**, E =** Eschar formation, **Ø:** Diameter of the recorded lesion in mm

P.ex. a 1mm papule in a slightly swollen area, with redness of 5mm diameter and minimal induration scores: **R2 (5mm), I1, S2, E1 (1mm)**

**Reaction Scoring Codes:**

**0 =** Absence/ no evidence of skin redness, swelling or induration or any lesion.

**Redness/erythema (Ø)**

**R1 =** Mild skin redness (pink skin in color)

**R2** = Moderate skin redness (red skin in color)

**R3** = Severe skin redness (dark red in color)

**Induration**

**I1 =** Mild induration (hardening) is palpated as a hard area with a diameter < 5 mm

**I2 =** Moderate induration, palpated as a larger, hardened area with a diameter 5 – 10 mm

**I3 =** Severe induration, the whole area is hardened and obviously enlarged with a diameter >10 mm

**Swelling/Edema**

**S1 =** skin slightly swollen, affected area is Ø < 5 mm

**S2 =** skin markedly swollen, the affected area is Ø 5 – 10 mm

**S3 =** skin obviously swollen, the affected area is Ø > 10 mm

**Eschar**

**E1 =** Indurated papule, color livid-deep red

**E2 =** Excoriated papule with small black scab/crust

**E3 =** Demarked excoriation with crust, raised indurated border

**Draize Score**

| **Score** | **Grade** | **Edema** | **Erythema** |
| --- | --- | --- | --- |
| 0 | None | no swelling | normal color |
| 1 | Minimal | Slight swelling; indistinct border | Light pink; indistinct |
| 2 | Mild | defined swelling; distinct border | bright pink/pale red; distinct |
| 3 | Moderate | defined swelling; raised border **(**<1mm**)** | bright red; distinct |
| 4 | Severe | pronounced swelling; raised border **(≥**1mm**)** | dark red; pronounced |
