# Supplementary material for: Characterization of the rhesus macaque (Macaca mulatta) scrub typhus model: Susceptibility to intradermal challenge with the human pathogen Orientia tsutsugamushi Karp
Source: PLoS Negl Trop Dis. 2018 Mar 9;12(3):e0006305. doi: 10.1371/journal.pntd.0006305 (PMC5862536; doi:10.1371/journal.pntd.0006305)
Supplement: S2 Table — Red blood cell (RBC), hematocrit (Hct), hemoglobin (Hb), white blood cell (WBC), mean red blood cell volume (MCV), mean red blood cell hemoglobin (MCH), and mean red blood cell hemoglobin concentration (MCHC). (DOCX) [file pntd.0006305.s002.docx]

**Table S2.** **Hematological values of control and *O. tsutsugamushi*-infected macaques.** Red blood cell (RBC), hematocrit (Hct), hemoglobin (Hb), white blood cell (WBC), mean red blood cell volume (MCV), mean red blood cell hemoglobin (MCH), and mean red blood cell hemoglobin concentration (MCHC).

| **Characteristics** | **Animal group** | **Day 0** | **Day 14** | **Day 28** |
| --- | --- | --- | --- | --- |
| RBC (10^6^/ml) | Control | 5.43±0.29 | 5.45±0.23 | 5.38±0.20 |
|  | Ot-infected | 5.79±0.25 | 4.91±0.19 | 5.53±0.28 |
| Hct (%) | Control | 38.83±1.49 | 38.97±1.21 | 38.47±1.03 |
|  | Ot-infected | 40.20±1.66 | 34.78±1.31 | 38.55±1.45 |
| Hb (gm%) | Control | 12.53±0.44 | 12.67±0.35 | 12.47±0.27 |
|  | Ot-infected | 13.15±0.61 | 10.95±0.49 | 12.28±0.50 |
| WBC (cells/ml) | Control | 7,627±326 | 6,053±647 | 7,310±772 |
|  | Ot-infected | 9,375±1,337 | ***10,375±1147 **** | 8,130±1256 |
| Platelet (cells/ml) | Control | 393,333±2,2615 | 375,667±4,2834 | 362,333±3,2824 |
|  | Ot-infected | 380,000±3,1830 | 357,500±3,3785 | 438,000±4,0156 |
| MCV (fl) | Control | 71.60±1.00 | 71.60±0.85 | 71.50±0.81 |
|  | Ot-infected | 69.45±0.88 | 70.85±1.06 | 69.85±1.43 |
| MCH (pg) | Control | 23.10±0.44 | 23.30±0.36 | 23.17±0.35 |
|  | Ot-infected | 22.70±0.29 | 22.28±0.32 | 22.23±0.37 |
| MCHC (g/dl) | Control | 32.30±0.25 | 32.53±0.13 | 32.40±0.15 |
|  | Ot-infected | 32.70±0.20 | 31.48±0.38 | 31.85±0.26 |

**Significant difference to control macaques (p<0.05)*
