# Supplementary material for: Characterization of the rhesus macaque (Macaca mulatta) scrub typhus model: Susceptibility to intradermal challenge with the human pathogen Orientia tsutsugamushi Karp
Source: PLoS Negl Trop Dis. 2018 Mar 9;12(3):e0006305. doi: 10.1371/journal.pntd.0006305 (PMC5862536; doi:10.1371/journal.pntd.0006305)
Supplement: S3 Table — Albumin (ALB), alkaline phosphatase (ALP), alanine transaminase (ALT), aspartate transaminase (AST), liver enzyme levels, were significantly raised at day 14, when compared to control macaques—but not creatinine, cholesterol, total bilirubin (TBIL), urea nitrogen, and creatinine kinase (CPK) levels. (DOCX) [file pntd.0006305.s003.docx]

**Table S3.** **Biochemical values of control and *O. tsutsugamushi*-infected macaques.**

Albumin (ALB), alkaline phosphatase (ALP), alanine transaminase (ALT), aspartate transaminase (AST), liver enzyme levels, were significantly raised at day 14, when compared to control macaques - but not creatinine, cholesterol, total bilirubin (TBIL), urea nitrogen, and creatinine kinase (CPK) levels.

| Characteristics | Animal group | Day 0 | Day 14 | Day 28 |
| --- | --- | --- | --- | --- |
| ALB  (g/dl) | Control | 4.10±0.00 | 4.10±0.15 | 3.97±0.12 |
|  | Ot-infected | 4.28±0.12 | ***3.25±0.12**** | 3.75±0.10 |
| ALP  (U/l) | Control | 389.90±58.62 | 435.20±97.10 | 416.60±101.70 |
|  | Ot-infected | 397.70±122.80 | ***234.60±46.07**** | 310.30±72.38 |
| ALT  (U/l) | Control | 46.07±10.21 | 42.73±7.43 | 35.80±7.17 |
|  | Ot-infected | 36.73±2.78 | ***27.95±4.50**** | 36.00±3.22 |
| AST  (U/l) | Control | 24.43±2.21 | 26.57±2.90 | 21.57±1.91 |
|  | Ot-infected | 23.30±1.54 | ***38.15±5.64**** | 25.78±1.88 |
| CA  (mg/dl) | Control | 9.63±0.22 | 9.22±0.13 | 9.97±0.14 |
|  | Ot-infected | 9.82±0.23 | 9.16±0.09 | 9.86±0.12 |
| CRE  (mg/dl) | Control | 0.66±0.02 | 0.62±0.00 | 0.60±0.02 |
|  | Ot-infected | 0.65±0.03 | 0.70±0.06 | 0.55±0.04 |
| CPK (U/l) | Control | 343.60±95.27 | 595.60±209.20 | 660.10±200.60 |
|  | Ot-infected | 290.30±39.30 | 698.50±97.04 | 469.40±36.67 |
| TBIL  (mg/dl) | Control | 0.14±0.01 | 0.13±0.02 | 0.10±0.02 |
|  | Ot-infected | 0.17±0.02 | 0.14±0.02 | 0.10±0.01 |
| TCHO  (mg/dl) | Control | 153.70±4.16 | 153.20±7.77 | 162.90±9.72 |
|  | Ot-infected | 130.90±7.13 | 110.10±2.42 | 137.90±10.40 |
| TP  (g/dl) | Control | 6.67±0.03 | 6.60±0.17 | 6.43±0.15 |
|  | Ot-infected | 6.95±0.12 | 6.73±0.14 | 6.93±0.17 |
| K^+^  (mEq/dl) | Control | 3.22±0.04 | 3.08±0.03 | 3.13±0.11 |
|  | Ot-infected | 3.11±0.09 | 3.31±0.08 | 2.93±0.06 |
| Na^+^  (mEq/dl) | Control | 142.90±1.47 | 142.50±1.42 | 141.60±1.02 |
|  | Ot-infected | 140.10±0.90 | 139.90±1.80 | 141.90±0.30 |
| Cl^-^  (mEq/dl) | Control | 109.50±1.32 | 108.20±1.02 | 108.40±1.12 |
|  | Ot-infected | 108.20±0.45 | 105.50±1.80 | 107.70±0.91 |

**Significant difference to control macaques (p<0.05)*
